# Gout In Extremis: Massive Soft Tissue Tophaceous Deposits

**DOI:** 10.1007/s11606-018-4662-9

**Published:** 2018-10-03

**Authors:** Melissa Rosas, Paul Aronowitz

**Affiliations:** Department of Internal Medicine, School of Medicine, University of California, Davis, Sacramento, CA USA

**Keywords:** rheumatology, clinical image, gout, tophi

**Figure 1:**
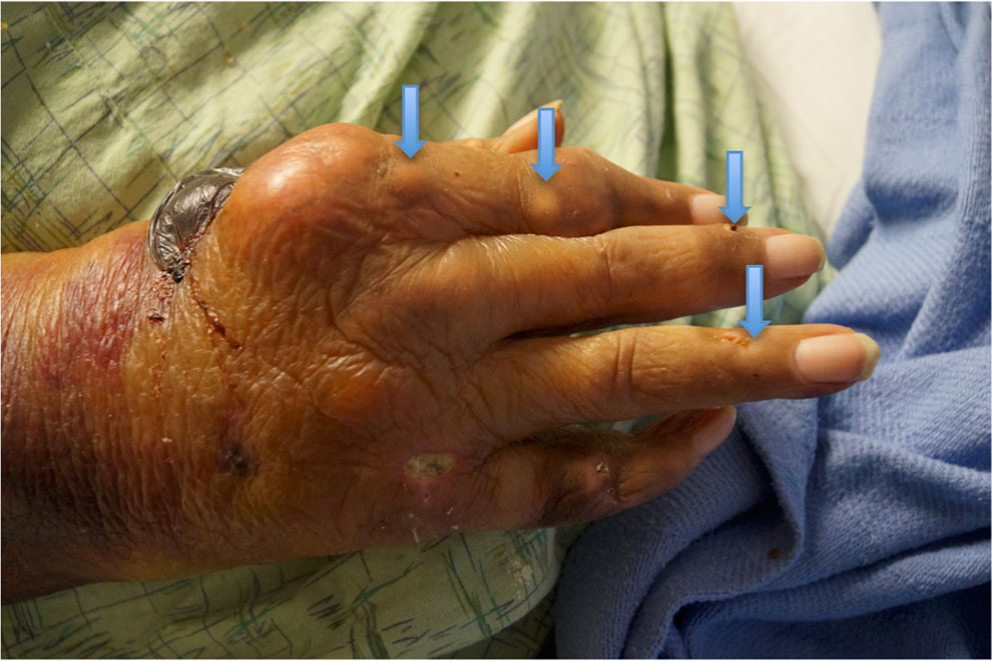
Tophaceous gout disfiguring the digits of the hand with tophi (arrows).

**Figure 2:**
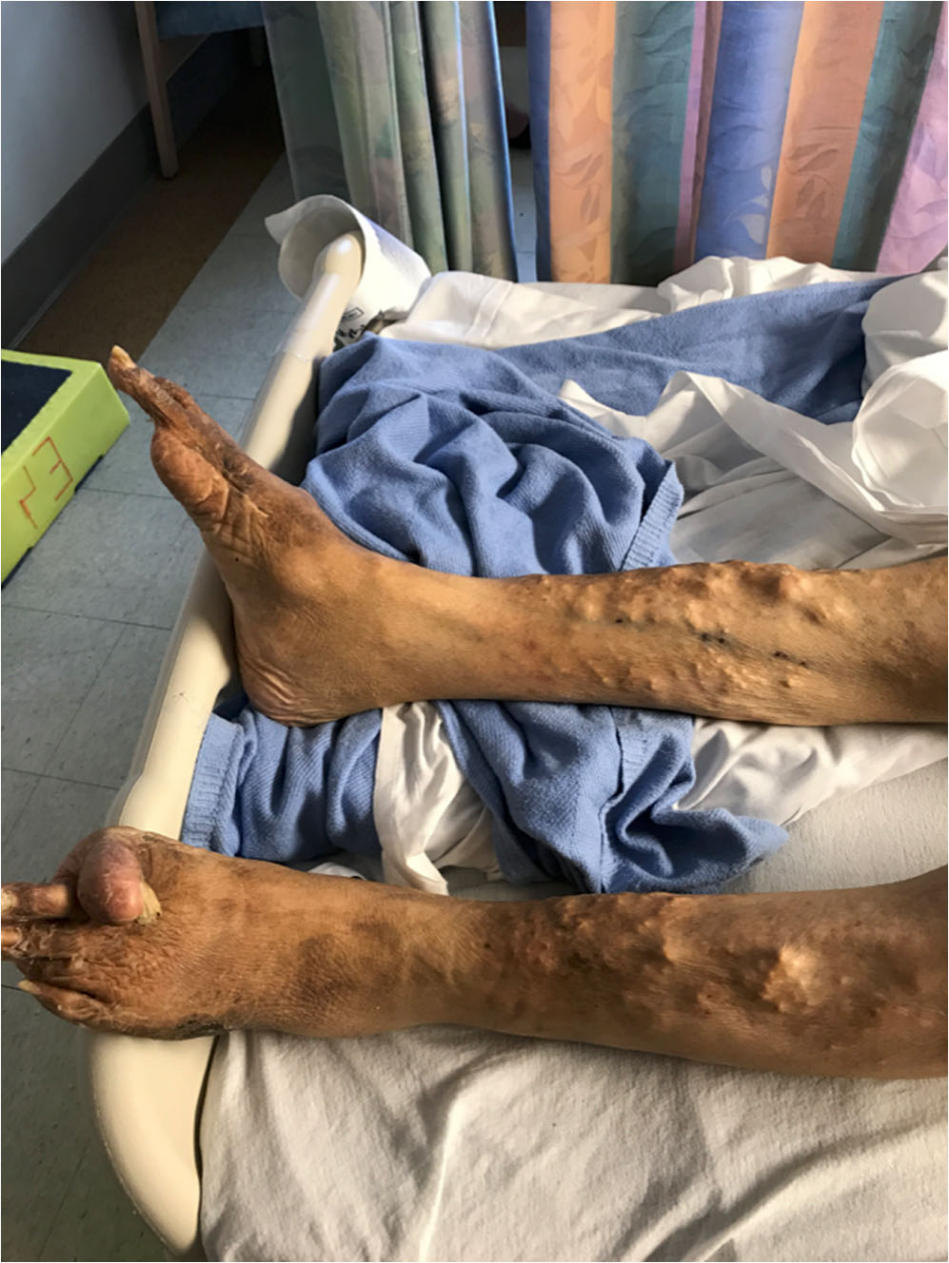
Tophaceous gout involving the bilateral anterior shins.

A 57-year-old Latino man with poorly controlled gout presented with severe, diffuse, bilateral hand pain and increasing drainage of white material from his hands. Physical examination revealed disfigured hands and feet with many tophi with areas of open skin draining a thick, white material (Fig. 1) and bilateral anterior shins with gouty tophi (Fig. 2). Laboratory studies revealed a uric acid level of 9.5 mg/dL.

Gout is an inflammatory arthritis. The first phase of gout is characterized by intermittent acute attacks with asymptomatic periods in between. If the gouty attacks and hyperuricemia are not properly treated over many years, the second phase of the disease, chronic tophaceous gout, develops. This phase manifests as polyarticular attacks, deposition of tophi in soft tissues and joints, and gout symptoms between attacks.^1^ Tophaceous gout can form soft tissue calcifications anywhere in the musculoskeletal system, including within tendons and extremities.^2^ The diagnosis of tophaceous gout is usually made by visual recognition of characteristic tophi, but rarely when the diagnosis is uncertain, synovial fluid or tophus aspiration, looking for negatively birefringent monosodium urate crystals can be performed. Plain radiography, ultrasound, computed tomography, or magnetic resonance imaging can identify structural changes related to this chronic inflammatory condition.^3^
